# Vitamin D and osteoporosis in chronic kidney disease

**DOI:** 10.1007/s40620-017-0430-x

**Published:** 2017-09-23

**Authors:** Paul Lips, David Goldsmith, Renate de Jongh

**Affiliations:** 1 Endocrine Section, Department of Internal Medicine, VU University Medical Center, P.O. Box 7057, 1007 MB Amsterdam, The Netherlands; 2 Cardiovascular and Cell Sciences Institute, St George’s University of London, Guy’s Hospital, London, UK

**Keywords:** Vitamin D, Osteoporosis, Bone mineral density, Chronic kidney disease

## Abstract

Osteoporotic fractures are common in patients with chronic kidney disease (CKD). Morbidity and mortality are higher in CKD patients with a fracture than in the general population. The assessment of bone mineral density for fracture prediction may be useful at all CKD stages. It should be considered when this influences treatment decisions. Vitamin D deficiency is common in patients with CKD, particularly in patients with proteinuria, due to loss of 25-hydroxyvitamin D and its binding protein. Vitamin D supplementation should be prescribed early in the course of renal disease. For treatment and prevention of vitamin D deficiency in CKD patients cholecalciferol 800 IU/day or the equivalent per month is recommended just as in the general population.

## Introduction

Chronic kidney disease (CKD) typically leads to profound changes in bone and calcium metabolism, resulting in renal osteodystrophy [1], predominantly osteitis fibrosa, osteomalacia, adynamic bone disease or a mixed variant. Over the last few decades the histological picture has been refined and has evolved, and a new classification has been proposed [2]. Metabolic bone disease with chronic kidney disease (CKD-MBD) may lead to osteoporosis, bone fragility and fractures. Fracture risk may be more than double in CKD compared to the normal population. CKD is a disease predominantly of old age, as of course is osteoporosis.

A further contributory factor to metabolic derangement and fracture risk is the ubiquity of vitamin D insufficiency and deficiency in CKD; at its simplest, this is evidenced by low serum concentrations of 25-hydroxyvitamin D [3]. With progressive loss of glomerular filtration rate (GFR) the serum concentration of the active vitamin D metabolite, 1,25-dihydroxyvitamin D, also falls. This may play a role in the pathogenesis of osteoporosis in patients with CKD. In this paper, we discuss the occurrence of osteoporotic fractures and the utility of bone mineral density (BMD) assessment in older persons without and with CKD. We also discuss vitamin D deficiency and its possible medical consequences in the “normal” population. The last aspect dealt with in this paper is vitamin D status in CKD. Recommendations on vitamin D supplementation in CKD will be given.

## Pathophysiology of osteoporosis in CKD

The imbalance between bone resorption and formation with ageing results in thinning of cortical bone, and thinning and disappearance of trabeculae in the cancellous bone, and these are the hallmark features of osteoporosis. In the bone disease of CKD, previously named renal osteodystrophy (and now CKD-MBD), bone “ageing” may be accelerated, leading to premature osteoporosis. Due to the changes in calcium and vitamin D metabolism, mineralization defects may occur, sometimes leading to osteomalacia. Usually, the rate of bone resorption and bone formation is increased, a consequence of secondary hyperparathyroidism, i.e. high bone turnover, leading to the histological picture of osteitis fibrosa, which is usually accompanied by, in particular, cortical bone loss. In patients with CKD, a mixed picture often is visible in bone biopsies. This indicates a diversity in potential contributing pathophysiological mechanisms resulting in significant heterogeneity in the bone abnormalities in CKD.

## Osteoporosis and fracture risk in patients with CKD and renal replacement therapy

It is well known that patients with CKD stages 3a-5D have increased fracture rates compared to the general population [4–7]. A prospective study on 10,955 participants from the atherosclerosis risk in communities (ARIC) study demonstrated a higher risk for fracture if the estimated GFR (eGFR) was below ≤60 ml/min/1.73 m^2^ [4]. A retrospective analysis of 2096 patients on renal replacement therapy showed that the symptomatic fracture risk in the renal replacement therapy population appeared to be twice as high in hemodialysis patients as in renal transplantation patients, while peritoneal dialysis patients had an intermediate risk [4]. The consequences of fractures may be more severe in CKD patients. Hospital stay duration in patients with hip fractures was 7 days in patients with end-stage renal disease (ESRD) compared to 5 days in patients without CKD, and the mortality was 5.9% in ESRD, 3.7% in CKD and 1.6% in patients with normal renal function [8].

In the last few years, several studies have demonstrated the predictive value of BMD, in particular of the femoral neck, for the occurrence of fractures in a wide range of CKD severity ranging from stage 3 to 5D [9–12]. In the earliest study, in 485 Japanese hemodialysis patients, lower femoral neck and total hip BMD predicted increased fracture risk. This predictive value, however, was higher in patients with parathyroid hormone (PTH) concentrations below the median of 204 pg/ml (21.6 pmol/l) as compared to those with PTH concentrations above the median [9]. Thus, BMD measurement is useful for fracture risk prediction in CKD stage 3 to 5D, probably particularly in those with low PTH concentrations. Also in a large prospective study in 2107 adults aged 40 years and older including 320 individuals with an eGFR ≤60 ml/min/1.73 m^2^ the fracture risk assessment tool (FRAX) with BMD, FRAX without BMD, and the femoral neck T-score all predicted fractures. The predictive value of FRAX was comparable between those with and without CKD, indicating a potential future role of the FRAX score in the prediction of fractures in CKD [10].

## Vitamin D deficiency and its consequences in the normal population

The main action of vitamin D is the stimulation of calcium absorption by the gut. The active metabolite 1,25(OH)2D stimulates the synthesis of calcium binding protein, calbindin-9K, and the opening of the calcium channels (TRPV5 and 6) in intestinal cells [13]. Bone mineralization is a passive process and it occurs when sufficient calcium and phosphate are available in the extracellular space. In case of vitamin D deficiency, the decrease of calcium absorption is compensated for by an increase in PTH, which stimulates the conversion of 25(OH)D into 1,25(OH)2D. Secondary hyperparathyroidism increases bone turnover, leading to bone loss [14]. Mild to moderate vitamin D deficiency may be one of the multiple causes of osteoporosis. It is common practice to prescribe vitamin D supplements to all patients with osteoporosis, and almost all randomized clinical trials with bone-sparing drugs have been performed with vitamin D (and sometimes calcium) supplements as adjunctive treatment. Severe vitamin D deficiency causes mineralization defects, and the clinical picture is that of rickets in children and osteomalacia in adults [14]. In addition, low-dose vitamin D supplementation may prevent falls and fractures, although very high doses may result in the opposite (see below).

Vitamin D status is usually assessed by the measurement of the serum 25(OH)D concentration either by immunochemical methods or by liquid chromatography followed by tandem mass spectrometry (LC-MS/MS), the latter being currently the gold standard [15]. However, assays for serum 25(OH)D vary considerably, and this may have a high impact on clinical decision making. The vitamin D external quality assessment scheme (DEQAS) showed variations of 10–20% below or above the mean and this resulted in differences in the part of the population to treat of more than 15% [16]. Quality control of assays for serum 25(OH)D is carried out by DEQAS and more recently by the vitamin D standardization program (VDSP). The latter used standard sera from the National Institute of Standardization Technology to improve accuracy [17].

A serum 25(OH)D level below 30 nmol/l indicates vitamin D deficiency [18]. According to the Institute of Medicine, the serum 25(OH)D should be above 50 nmol/l for optimal bone health [18], though it is not known if this applies in CKD. The vitamin D status has been assessed in most countries of the world with a wide variation of latitudes. Serum 25(OH)D was assessed in the Longitudinal Aging Study Amsterdam, an ongoing cohort study in the older population >55 years old. In the group >65 years, serum 25(OH)D was lower than 50 nmol/l in 48% of the population [19]. In the bazedoxifene clinical trial, vitamin D status was assessed at baseline in more than 7000 postmenopausal women from seven continents with a high risk of osteoporosis. Serum 25(OH)D was assessed in a central laboratory. About 50% of the population at >40° latitude had a serum 25(OH)D < 50 nmol/l at least in winter [20].

Mineralization defects in transiliac bone biopsies of patients with hip fracture were visible when serum 25(OH)D was lower than 30 nmol/l [21] although optimal mineralization occurs when serum 25(OH)D increases to above 50 nmol/l. In the LASA study, a significant positive relationship was observed between serum 25(OH)D and BMD of the hip up to a threshold of serum 25(OH)D of about 50 nmol/l [22]. A vitamin D supplementation study with 400 IU/day vs. placebo in 2578 persons aged >65 years showed a mean increase of femoral neck BMD of more than 2% in 2 years [23]. However, this did not result in a decrease of the incidence of hip and other fractures [24]. A study in 3270 nursing home residents in Lyon, France with vitamin D 800 IU/day with calcium 1200 mg/day vs. double placebo resulted in a significant decrease of the incidence of hip and other fractures [25]. Altogether, 19 randomized clinical trials with vitamin D ± calcium vs. placebo have been performed. Of these, five trials showed a decrease of fracture incidence, one showed a borderline decrease, one a decrease in the per protocol analysis and ten trials did not show an effect. However, in two trials an increase of fracture incidence was observed [26, 27]. These studies used a very high dose once per year, leading to very high serum 25(OH)D concentrations during the first few months after the bolus dose [28]. Meta-analyses usually agree that vitamin D is more effective for fracture prevention: (1) in institutionalized than community-living elderly, (2) with a dose of ≥800 IU/day than with a lower dose, (3) in 80+ and 70–80 year-old persons than in younger persons, and (4) when compliance is higher than 80% [29].

## Vitamin D status in CKD and vitamin D supplementation

Vitamin D deficiency is common in patients with CKD. In a cross-sectional study in 201 patients with a calculated GFR of 27 ± 11 ml/min and a mean age of 65 years, serum 25(OH)D was 48 ± 34 nmol/l [3]. In another study, vitamin D deficiency was associated with a greater risk of proteinuria, ESRD, coronary calcification and mortality [30]. However, it might be that there is reverse causality, i.e. the proteinuria might cause the vitamin D deficiency, explained by a greater loss of 25(OH)D bound to vitamin D binding protein. In the study of Gonzalez et al. [31], serum 25(OH)D was much lower in CKD patients with proteinuria than in those without. In this study, a very significant positive correlation was found between serum 25(OH)D and serum 1,25(OH)2D, a consequence of substrate dependent synthesis of 1,25(OH)2D during vitamin D deficiency. In the Korean National Health and Nutrition Examination Survey, BMD was lower in patients with CKD, but within the CKD group the BMD was lower in those with serum 25(OH)D < 50 nmol/l than in those with serum 25(OH)D > 50 nmol/l [32]. In patients with hemodialysis renal osteodystrophy, serum 25(OH)D concentrations below 50 nmol/l were associated with an increase in bone turnover as assessed with bone biopsies [33]. The 2009 Kidney Disease Improving Global Outcomes (KDIGO) CKD-MBD guideline does not make a recommendation whether and when to measure serum 25(OH)D and on the required serum level of 25(OH)D in CKD patients. For supplements it suggests to conform to the guideline for the general population. The Institute of Medicine recommends cholecalciferol 600 IU/day for adults and 800 IU/day for all people aged 70 years and older [18].

A systematic review and meta-analysis of observational studies and randomized clinical trials of vitamin D supplementation in CKD concluded that a significant increase in serum 25(OH)D occurred as well as a significant decline in serum PTH. This was associated with a low incidence of hypercalcemia and hyperphosphatemia during vitamin D supplementation, which resolved after discontinuation [34]. This may have been due to the high doses used in the clinical trials.

## Conclusions

Osteoporotic fractures are very common in patients with CKD, but age also plays an important role, and of course ageing and osteoporosis are closely linked. Morbidity and mortality following fracture are higher in CKD patients than in the general population. The assessment of bone mineral density for fracture prediction may be useful in all CKD stages. It should be considered when its results influence treatment decisions. Vitamin D deficiency is common in patients with CKD, particularly in patients with proteinuria, due to loss of 25-hydroxyvitamin D with vitamin D binding protein. The advantage of native vitamin D is maintenance of feedback mechanisms and possible effects due to extrarenal hydroxylation. Vitamin D supplementation should be prescribed early in the course of renal disease. For treatment and prevention of vitamin D deficiency in CKD patients cholecalciferol 800 IU/day or the equivalent per month is recommended just as in the general population.
